# 4-Hydroxyphenylpyruvate Dioxygenase-Like predicts the prognosis and the immunotherapy response of cancers: a pan-cancer analysis

**DOI:** 10.18632/aging.205591

**Published:** 2024-03-06

**Authors:** Huimin Li, Junzhi Liu, Shurui Wang, Yue Xu, Qiang Tang, Guoguang Ying

**Affiliations:** 1 Laboratory of Cancer Cell Biology, Tianjin Medical University Cancer Institute and Hospital, National Clinical Research Center for Cancer, Tianjin 300060, China; 2 Department of Otorhinolaryngology, Tianjin Medical University General Hospital, Tianjin 300070, China; 3 Chinese Academy of Medical Sciences and Peking Union Medical College, Beijing 100730, China; 4 The Second Affiliated Hospital of Zhejiang University School Medicine, Hang Zhou 310000, China

**Keywords:** 4-Hydroxyphenylpyruvate Dioxygenase-Like, pan-cancer, prognostic biomarker, immunotherapy response, proliferation

## Abstract

The 4-Hydroxyphenylpyruvate Dioxygenase-Like (HPDL) protein plays a crucial role in safeguarding cells from oxidative stress by orchestrating metabolic reprogramming. New research suggests that HPDL is considerably increased in pancreatic ductal adenocarcinoma, although its impact on cancer immunotherapy is still unclear. Pancancer transcriptional data were obtained from The Cancer Genome Atlas (TCGA) and the Genotype-Tissue Expression datasets. The cBioPortal webtool was utilized to examine genomic changes in different cancer types. The prognostic significance of HPDL in pancancer was evaluated using univariate Cox regression analysis. Extensive utilization of the CTRP and PRISM databases was performed to forecast potential medications that specifically target HPDL in LUAD. In summary, studies were conducted to evaluate the impact of HPDL on the proliferation and movement of LUAD cells using loss-of-function experiments. HPDL is expressed excessively in a wide variety of cancer types, indicating its prognostic and predictive value. Moreover, we emphasized the strong correlation between HPDL and indicators of immune stimulation, infiltration of immune cells, and expression of immunoregulators. The remarkable finding of the HPDL was its capacity to precisely anticipate responses to cancer therapies using anti-PDL1 and anti-PD1 antibodies among individuals. Moreover, HPDL can function as a predictive marker for specific inhibitors in instances of cancer. Suppression of HPDL resulted in reduced growth and movement of LUAD cells. To summarize, our results suggest that HPDL acts as a prospective predictor of outcomes and a positive indication of response to immunotherapy in patients undergoing treatment with immune checkpoint inhibitors (ICIs).

## INTRODUCTION

The reprogramming of energy metabolism, which facilitates rapid cellular growth and proliferation through the adjustment of energy metabolism pathways, has emerged as a defining characteristic of cancer [1]. Due to their heterogeneity and complex structure, tumor cells engage in diverse metabolic pathways. They not only metabolize glucose to generate ATP but also utilize other substrates such as glutamine, serine, arginine, fatty acids, and various lipid compounds to fuel their rapid proliferation [2]. According to recent research, HPDL protects cells from oxidative stress by changing the metabolic profile of cancer cells, giving priority to glutamine metabolism [3]. Undoubtedly, the crucial function of the metabolic process of glutamine is to enhance immune cells and control the transformation of CD4+ T cells into proinflammatory subtypes [4, 5]. Different immune cells have shown an association between their functional activity and increased utilization of glutamine. These activities encompass cellular proliferation, display of antigens, generation and secretion of cytokines, generation of nitric oxide and peroxide, and engulfment of foreign particles. The availability of NADPH reserves is crucial for all these processes [6].

The three-dimensional growth of cell lines associated with pancreatic ductal adenocarcinoma (PDAC) is influenced by HPDL, a protein found in the intermembrane space of mitochondria. Moreover, it contributes to the stimulation of MIAPACA2 orthotopic xenograft growth [6, 7]. The overproduction of HPDL also enhances the growth of PDAC *in vitro* [3]. Research showed that the removal of HPDL did not have a notable impact on the development of orthotopic or subcutaneous PATU-8902 tumors [7]. Other routes for producing the CoQ10 headgroup or the removal of CoQ10 or its biosynthetic intermediates could be the cause for this. PDAC patients exhibiting elevated HPDL expression face poorer overall survival rates. Consequently, therapies specifically targeting HPDL or its associated CoQ10 biosynthetic pathways could offer substantial benefits for this patient group. However, a comprehensive understanding of tumor dependence on HPDL heterogeneity is needed before such therapies can prove effective. Various clinical pathologies can occur due to a shortage of COQ enzymes, and a lack of CoQ10 can lead to a deficiency [8]. Recently, connections have been made between differences in HPDL and disorders such as childhood spastic cerebral palsy and significant impairments in neurodevelopment, as well as deficiencies in myelination [9–12]. Mice lacking HPDL showed seizures, reduced brain size due to apoptosis, and died shortly after birth, resembling the neurodegenerative condition observed in individuals with HPDL mutations [11]. Our research indicates that HPDL promotes tumor growth, migration and cell cycle in LUAD cells, and the administration of 4-Hydroxymethyl-2-furfural (4-HMA), 4-Hydroxybenzoic acid (4-HB), or Coenzyme Q10 (CoQ10) to patients bearing HPDL mutations may serve to ameliorate or stabilize certain symptomatic manifestations.

Observing the aberrant expression of HPDL in cancer led us to hypothesize that genetic alterations in HPDL might be responsible for this phenomenon. To investigate this, we analyzed the genetic alterations of HPDL in TCGA Pancancer tumor samples. The objective of this study was to reveal the differences in HPDL expression between malignant tissues and normal human tissues by analyzing transcriptomic data from the TCGA-Pancancer cohort and GTEx dataset. The significance of HPDL in predicting cancer was evaluated using the univariate Cox regression technique for all types of cancer. GSEA revealed the cancer characteristics associated with HPDL expression. Various methods, such as CIBERSORT, XCELL, MCPCounter, EPIC, and TIDE, were utilized to assess the abundance of immune cells in cancer specimens. The associations between the infiltration of immune cells and the expression of HPDL were computed using these methods in every instance of cancer. Finally, two separate groups of cancer patients who received immune checkpoint blockade (ICB) treatment were used to evaluate the effectiveness of HPDL in predicting outcomes and determining the response to immunotherapy.

## MATERIALS AND METHODS

### Data source and processing

Transcriptional data of cancer tissue were provided by the TCGA-Pancancer group, whereas normal human tissue data were supplied by the Genotype-Tissue Expression (GTEx) database. Both datasets were acquired through UCSC Xena [13] (https://xenabrowser.net/). The expression profiles were converted to transcripts per kilobase million (TPM) format, and the data in log2(TPM+1) format were utilized for further analysis. Among the thirty-three cancer types, twenty-two had available data on normal tissue through the matched information obtained from the website Gene Expression Profiling Interactive Analysis 2 (GEPIA2; http://gepia2.cancer-pku.cn/#dataset) [14].

### Genomic alterations analysis of HPDL in human cancers

The cancer genomics database cBioPortal is capable of detecting molecular data in cancer tissues and comprehending the related genetics, epigenetics, gene expression, and proteome [15, 16]. For this study, we utilized cBioPortal to examine the frequencies of genetic modifications (such as mutations, structural variations, amplifications, deep deletions, and multiple alterations) in HPDL cells across various types of cancer. Moreover, we utilized bar graphs from the cBioPortal web-based application to exhibit the occurrence rate of genetic alterations.

### HPDL protein localization and interaction

The HPA (http://www.proteinatlas.org) showcases the protein expression patterns of various human tissue types [17]. To demonstrate the cellular distribution of HPDL in cancer cells, we utilized immunofluorescence pictures of three distinct human cancer cell lines (MCF7, U-20S, and CACO-2). ComPPI, available at https://comppi.linkgroup.hu/, is an innovative and open database that provides information on protein-protein interactions (PPIs). By combining data from various databases, this system offers extensive knowledge on interactions, proteins, and their specific locations [18]. The ComPPI website provided the protein-protein interaction (PPI) data for HPDL. The protein information was annotated by using the ‘ID mapping’ feature of the UniProt database, which can be accessed at https://www.uniprot.org/.

### Prognostic analysis

The UCSC Xena database also provided additional prognostic information, including overall survival (OS), progression-free survival (PFS), disease-free survival (DFS), and disease-specific survival (DSS). Next, univariate Cox regression analysis was conducted to evaluate the predictive significance of HPDL for a particular prognosis group in each type of cancer. In the univariate Cox regression analysis, the continuous variable expression data from the HPDL were utilized, and the cutoff was determined using the ‘surv-cutpoint’ function from the R package ‘survminer’ (version 0.4.9). In summary, the relative risk (RR) and its corresponding 95% confidence interval (CI) were calculated and displayed in a forest plot, which provides a visual representation of the results.

### Identification of differentially expressed genes (DEGs) between low- and high-HPDL subgroup

To identify the genes that exhibited differential expression in each form of cancer, the patients were categorized into two subgroups based on their HPDL levels: the high-HPDL subgroup, comprising the top 30%, and the low-HPDL subgroup, comprising the bottom 30%. To compare the low-HPDL and high-HPDL subgroups, we conducted differential expression analyses with the R package ‘limma’ [19]. We obtained the log2 (fold change) and adjusted P value for each gene in relation to all types of cancer. Differentially expressed genes (DEGs) were categorized as genes with P values less than 0.05. Supplementary Table 1 displays the differentially expressed genes (DEGs) between the low-HPDL subgroup and the high-HPDL subgroup for every cancer type (Supplementary Table 1).

### Gene set enrichment analyses

The file ‘h.all.v7.4.symbols.gmt’, containing 50 hallmark gene sets, was acquired from the Molecular Signatures Database (MSigDB, https://www.gsea-msigdb.org/gsea/index.jsp). Normalized Enrichment Score (NES) and False Discovery Rate (FDR) for each cancer type were calculated by using this file to analyze the biological processes associated with them. For each cancer type, we obtained the 20 most important HPDL pathways. To generate GSEA visualizations for the most dominant immune pathways in the top 8 cancer types, we employed the ‘ggridges’ tool available at https://wilkelab.org/ggridges/. The R software packages ‘clusterProfiler’ and ‘GSVA’ [20, 21] were employed to perform gene set enrichment analysis. The condensed findings are displayed in a bubble chart created using the R library ‘ggplot2’.

### Immune cell infiltration analysis of HPDL

The TIMER resource offers various techniques to analyze the infiltration of immune cells in different types of cancer, providing data [22]. To establish the associations between the infiltration of immune cells associated with HPDL and the TCGA Pancancer project, relevant data were obtained from the TIMER2.0 database (http://timer.cistrome.org/) by utilizing the ‘Gene’ function under the ‘Immune Association’ section. To create a heatmap showing the statistical Spearman correlations between HPDL mRNA expression and 19 various immune cell subsets in different types of cancer, we employed the R package ‘ggplot2’. The groups included cancer-related fibroblasts (CAFs), lymphoid precursors, B cells, neutrophils, hematopoietic stem cells (HSCs), CD4+ T cells, myeloid precursors, monocyte precursors, endothelial cells (Endos), eosinophils (Eos), regulatory T cells (Tregs), follicular helper T cells, NK T cells, g/d T cells, monocytes, macrophages, dendritic cells, CD8+ T cells, mast cells, and NK cells.

### Immunotherapy prediction analysis

For each type of cancer, Spearman correlation analysis was conducted to determine the connections between HPDL and the documented cancer immunotherapy biomarkers. The correlation between HPDL and tumor mutational burden (TMB) as well as microsatellite instability (MSI) was examined in pancancer through Spearman correlation analysis. To investigate the correlation between HPDL and the efficacy of immune checkpoint blockade (ICB) therapy, two sets of ICB treatments were acquired to confirm the predictive capability of HPDL in the response to immunotherapy. In the IMvigor210 [7] study, 298 patients with urological cancer received atezolizumab (anti-PDL1), while the GSE78220 [23] dataset comprised transcriptomic profiles of 27 melanoma patients prior to nivolumab treatment (anti-PD1). The patients were categorized into two groups based on their HPDL expression levels: one group had low expression, while the other had high expression. The categorization was performed by utilizing the optimal threshold value with the ‘surv-cutpoint’ characteristic of the ‘survminer’ R package. To assess the disparity in response rates between the cancer groups with low-HPDL and high-HPDL, a chi-square test was employed.

### Potential sensitive drug prediction

A previous study was carried out to analyze the sensitivity of drugs [24]. Data on the sensitivity of human cancer cell lines were obtained from the Cancer Therapeutics Response Portal (CTRP v.2.0, https://portals.broadinstitute.org/ctrp) and the PRISM Repurposing dataset (19Q4, released December 2019, https://depmap.org/portal/prism/). The AUC value for each individual sample was computed utilizing the ‘oncoPredict’ software package [25]. Higher sensitivity to potential drugs is indicated by lower AUC values, as stated in reference [24]. In addition, we utilized the GDSC database to anticipate how LUAD patients would react to chemotherapy medications. The response of patients to chemotherapy drugs was evaluated by calculating the IC50, which represents the concentration at which half of the inhibitory effect is achieved, using the ‘OncoPredict’ program.

### Statistical analyses

To evaluate statistical significance and compare the levels of HPDL expression in tumor and normal tissues, the Wilcoxon rank-sum test was conducted. The prognostic significance of HPDL expression in all types of cancer was evaluated using univariate Cox regression analysis. The predictive value of HPDL expression in the ICB therapy groups was evaluated using the Kaplan-Meier method (log-rank test) to determine its significance. To assess the statistical associations between HPDL and various factors, such as immune cell infiltration levels, immune regulator genes, TMB, and MSI, a Spearman correlation analysis was conducted. In summary, the chi-square test was employed to establish the statistical significance of comparing the proportions of ICI therapy responders and nonresponders in the low-HPDL and high-HPDL cancer subcategories.

### Western blotting

The cells were lysed in lysis buffer at a low temperature, which included phosphatase and protease inhibitors. Protein concentrations were determined using the bicinchoninic acid assay. Protein samples were separated using 4–12% SDS/PAGE and then transferred onto PVDF membranes. After blocking and incubation with primary antibodies, the membranes were then incubated with secondary antibodies. Chemiluminescent solution was utilized to identify immunoreactive proteins.

### Cell lines and transfection

A549 and PC9 cells were cultivated in a humid atmosphere consisting of 5% CO_2_ at 37°C. The cells were grown in Roswell Park Memorial Institute (RPMI 1640, Gibco, United States) enriched with 10% FBS (HyClone, USA) and 1% penicillin-streptomycin. The shRNA plasmids were sold by Shanghai Jikai Gene Co., Ltd. The Lipofectamine^®^ 3000 kit was used to carry out transfection, following the guidelines provided by the manufacturer.

### Transwell assays

Transwell assays were utilized to assess cell migration. In the upper compartment, 3 × 10^5^ LUAD cells were introduced and suspended in 200 μL of FBS-free medium. To improve the lower chamber, a medium containing 10% FBS was introduced. Following a 24-hour incubation period, the cells that adhered to the lower membrane were immobilized and colored. Afterwards, a microscope was employed to determine the quantity of cells in six arbitrarily chosen areas.

### Cell viability and colony formation assays

The viability of LUAD cells was assessed using a Cell Counting Kit-8 (CCK8) assay (Yeasen, USA) at a wavelength of 450 nm after seeding 2.5 × 10^3^ cells per well in 96-well plates and evaluated at four different time points (24 h, 48 h, 72 h, and 96 h). We performed a colony formation experiment to analyze cellular proliferation. Following the preparation of separate cell suspensions, a total of 1200 cells were evenly allocated into every well of a 6-well plate. Following a period of 11–14 days, the cells were treated with 3.7% formaldehyde (Sigma-Aldrich, USA) and subsequently subjected to staining using 0.4% crystal violet (Solarbio, China). Following three immersions in fresh water, the plates were washed and subsequently scanned.

### Availability of data and materials

The open-access datasets are available through the following URL: GSE91061 (https://www.ncbi.nlm.nih.gov/geo/query/acc.cgi?acc=GSE91061), GSE78220 (https://www.ncbi.nlm.nih.gov/geo/query/acc.cgi?acc=GSE78220), and the Cancer Genome Atlas (TCGA) database.

## RESULTS

### HPDL exhibits aberrant expression in neoplastic tissues

**Figure 1:**
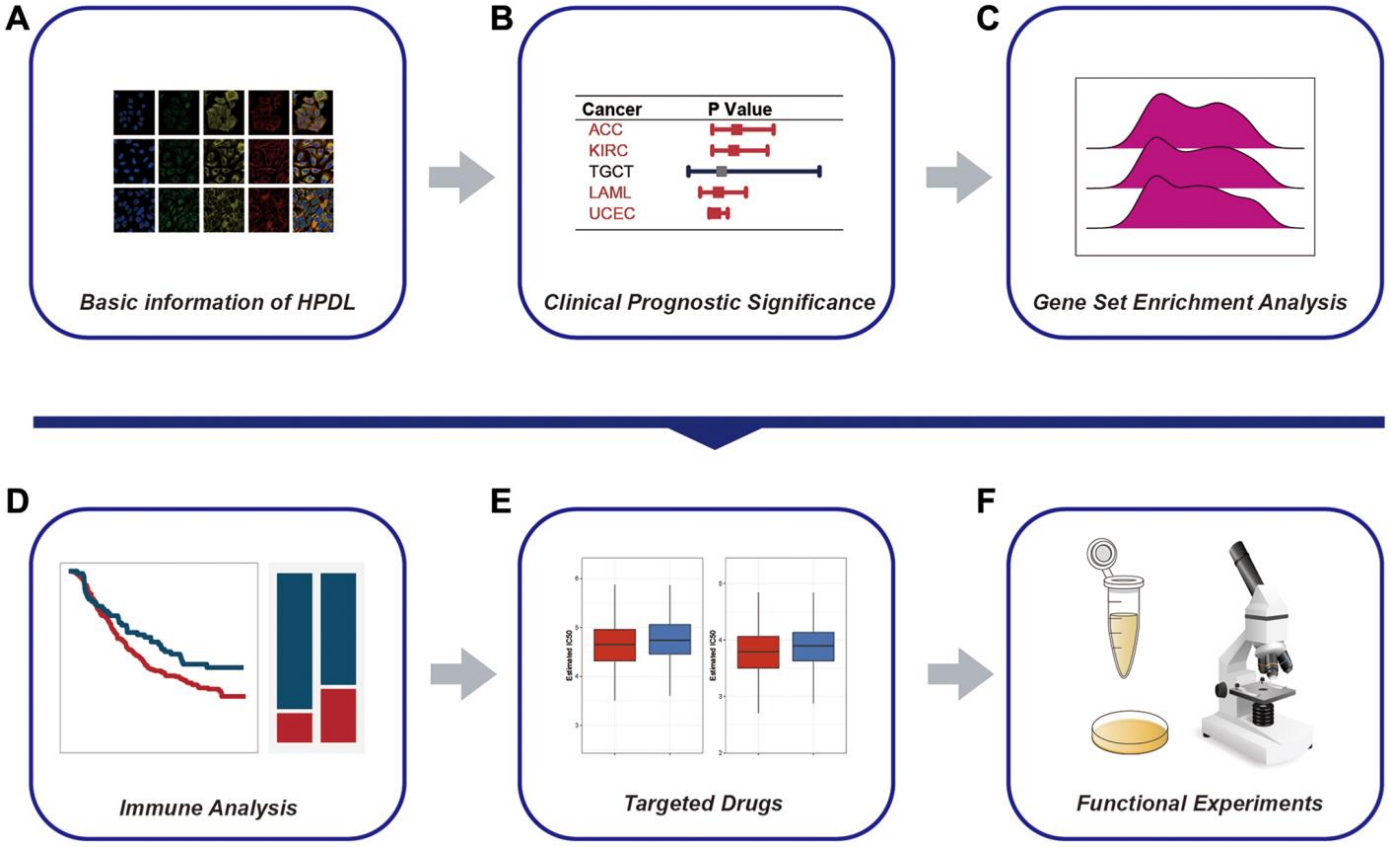
**The workflow of the study.** (**A**) Basic information of HPDL. (**B**) Clinical prognostic significance of HPDL in pancancer. (**C**) Gene set enrichment analysis of HPDL in pancancer. (**D**) Immune infiltration analysis of HPDL. (**E**) Identification of candidate drugs of HPDL. (**F**) The biological function of HPDL was verified by laboratory experiments.

**Figure 2:**
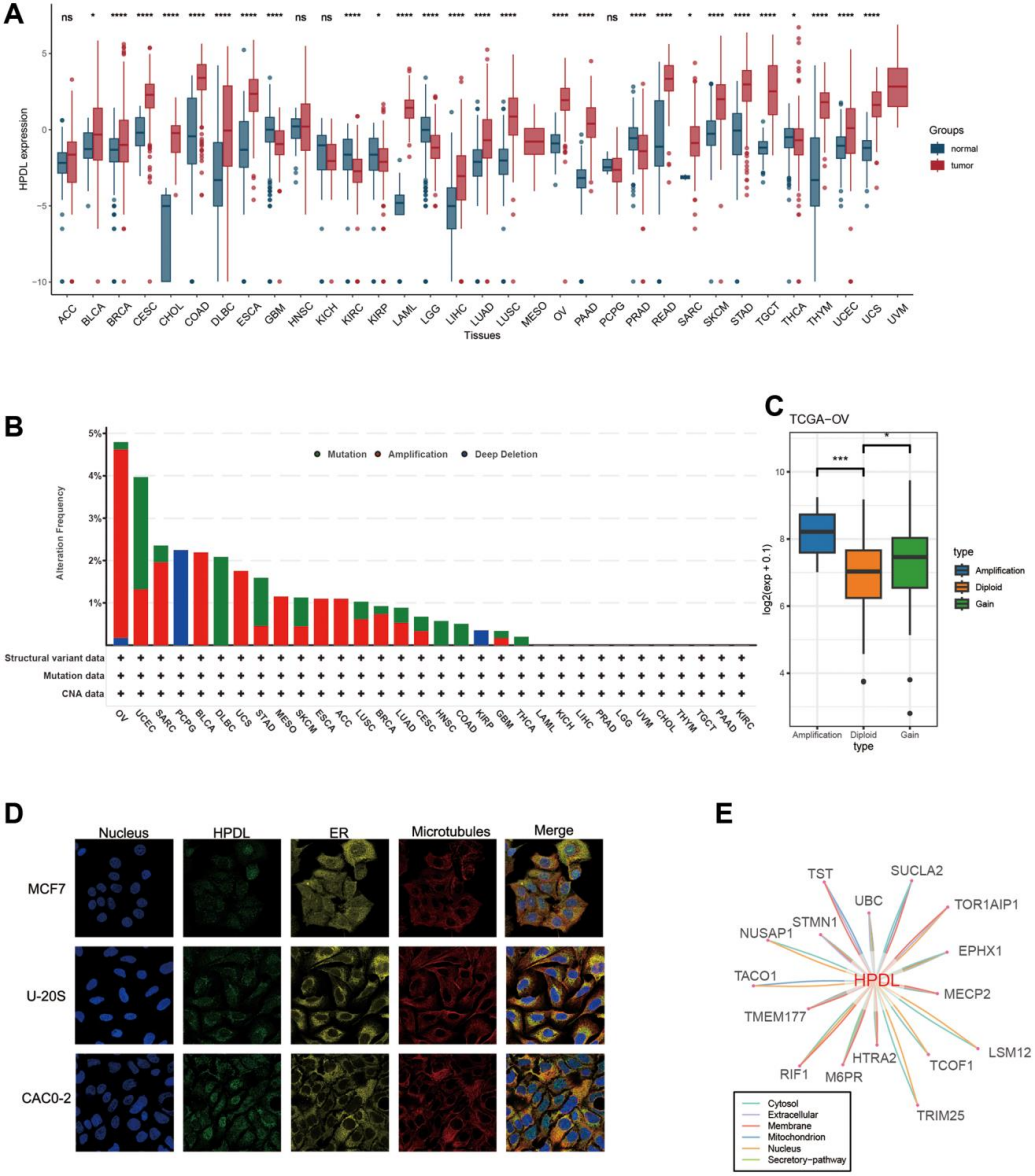
**Basic information of HPDL.** (**A**) The level of HPDL expression between tumor and normal tissues in each type of cancer based on the integrated data from TCGA and GTEx datasets. (**B**) Analysis of HPDL change frequency in pan-cancer research according to cBioPortal database. (**C**) The HPDL expression levels between HPDL-deletion and diploid OV patients. (**D**) The immunofluorescence images of HPDL protein, nucleus, endoplasmic reticulum (ER), microtubules and the incorporative images in MCF7, U20S, and CAC02 cell lines. (**E**) The protein-protein interaction (PPI) network presents the proteins interacting with HPDL. ^****^P < 0.0001, ^***^P < 0.001, ^**^P < 0.01, ^*^P < 0.05, ^ns^*P* > 0.05.

Figure 1 is the workflow of the study. To clarify the patterns of HPDL expression in different types of cancer, we combined TCGA databases and performed a comprehensive analysis on the levels of HPDL mRNA expression in a diverse range of malignant diseases. The results showed increased HPDL levels in 21 different types of tumors, such as BLCA, BRCA, CESC, CHOL, COAD, DLBC, ESCA, LAML, LIHC, LUAD, LUSC, OV, PAAD, READ, SARC, SKCM, STAD, TGCT, THYM, UCEC, and UCS. Conversely, reduced HPDL expression was detected in six tumors, specifically GBM, KIRC, KIRP, LGG, PRAD, and THCA (Figure 2A). Similar outcomes were identified in paired tissue samples. Remarkably, HPDL expression was markedly raised in PAAD, which may be attributable to its function in safeguarding cells from oxidative stress by promoting glutamine metabolism [3]. In line with prior research, these results indicated a discrepancy in HPDL expression in cancerous conditions.

### Genetic alteration proportions of HPDL remain below 5% in the majority of cancers

Considering the abnormal expression of HPDL in cancer, our hypothesis is that genetic changes in HPDL might play a role in this occurrence. As a result, we conducted a genetic analysis of HPDL by utilizing tumor samples obtained from TCGA Pancancer. Based on Figure 2B, the prevalence of ‘Amplification’ was the highest among patients diagnosed with ovarian serous cystadenocarcinoma (OV), surpassing 4% in frequency. We subsequently analyzed HPDL expression in the Amplification and diploid groups of OV tumors, discovering that expression in the Amplification group was notably higher compared to the Diploid group (Figure 2C). On the other hand, the uterine corpus primarily showed the ‘Mutation’ type of copy number alteration (CNA) in cases of endometrial carcinoma, with an alteration frequency of approximately 2.5% (Figure 2B). Significantly, HPDL Deep Deletion (Figure 2B) was observed in all PCPG cases with genetic mutations, accounting for approximately 2% of the overall occurrences. Generally, the occurrence of HPDL genetic alterations was not elevated, which may be associated with the highly conserved nature of HPDL.

### HPDL protein localization, interaction, and expression

To gain the HPDL protein, we obtained the immunofluorescence of HPDL protein images from the Human Protein Atlas (HPA), retrieved protein-protein interaction information from the ComPPI database. According to the information obtained from the HPA database (Figure 2D), analysis of immunofluorescence (IF) images revealed that the HPDL protein predominantly resided in the nucleoplasm of the MCF7, U20S, and CAC02 cancer cell lines. Subsequently, the PPI network was built by utilizing the interaction data obtained from the ComPPI platform. Figure 2E demonstrates the distribution of proteins that have close interactions with HPDL in different cellular compartments, including the cytosol, mitochondria, nucleus, extracellular space, secretory pathway, and membrane. These findings suggest that the HPDL protein is aberrantly expressed and possesses potential functional significance in cancers.

### Clinical prognostic significance of HPDL in pan-cancer

**Figure 3:**
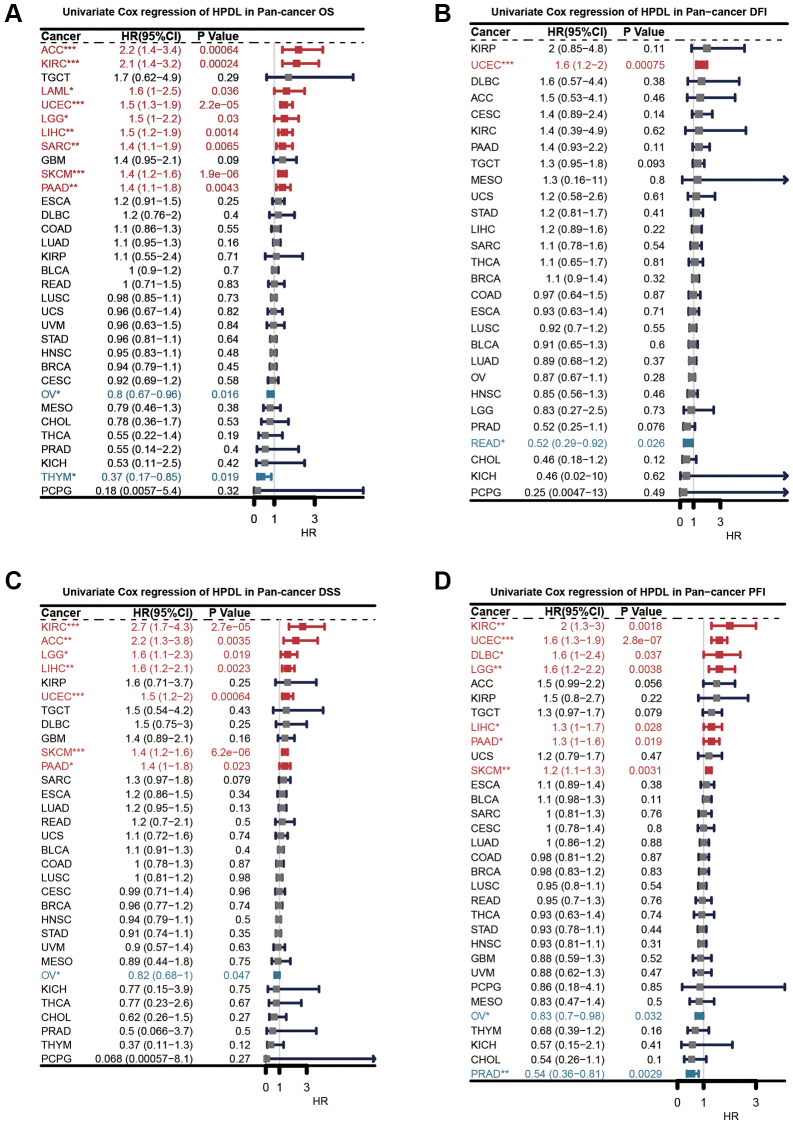
**Univariate Cox regression analysis of HPDL.** The results were shown with a forest map for (**A**) OS; (**B**) DFI; (**C**) DSS; (**D**) PFI.

Next, we carried out an extensive investigation into the predictive significance of HPDL in a comprehensive study encompassing various forms of malignancies. Individually, we assessed the rates of survival, including overall survival (OS, shown in Figure 3A), disease-free interval (DFI, illustrated in Figure 3B), disease-specific survival (DSS, depicted in Figure 3C), and progression-free interval (PFI, presented in Figure 3D). To reduce the impact of perplexing prejudices, the researchers utilized univariate Cox regression. First, HPDL acted as an overall survival predictive gene for the survival of ACC, KIRC, LAML, UCEC, LGG, LIHC, SARC, SKCM, PAAD, OV, and THYM. Further evaluation of disease-free survival demonstrated HPDL to be a standalone prognostic gene in UCEC and READ. HPDL emerged as a standalone prognostic gene in the examination of disease-specific survival across KIRC, ACC, LGG, LIHC, UCEC, SKCM, PAAD, and OV. The analysis of PFI revealed that HPDL acted as a standalone prognostic gene in KIRC, UCEC, DLBC, LGG, LIHC, PAAD, SKCM, OV, and PRAD. When the hazard ratio is greater than one, HPDL acts as a prognostic risk factor for poor survival in most cancer instances. The complexity and diversity in the prediction of cancer outcomes using HPDL varied across different types of cancer. Further inquiries should prioritize examining the involvement of the HPDL protein in cancerous cells.

### Gene set enrichment analysis of HPDL reveals its association with cancer immune response

**Figure 4:**
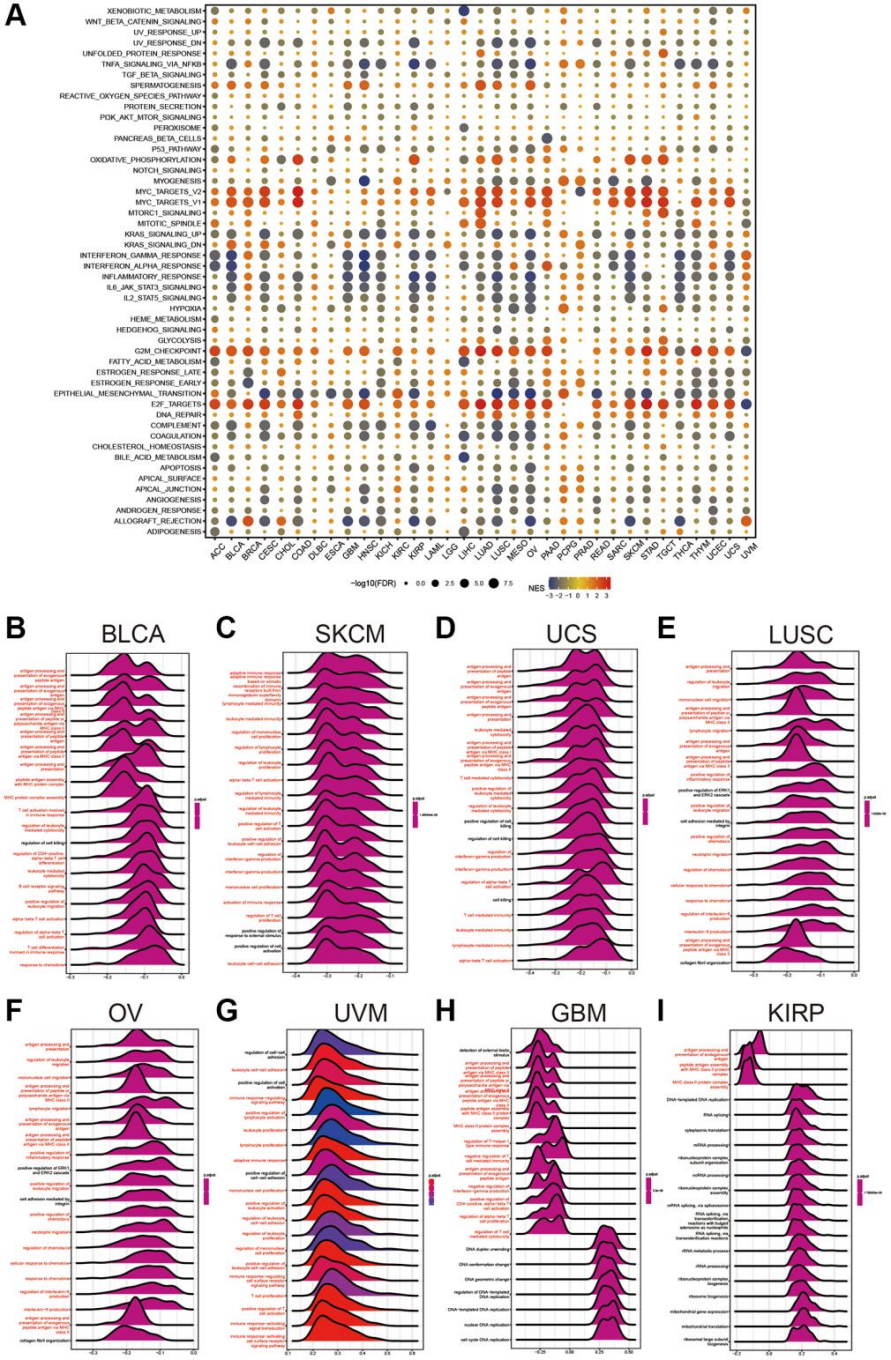
**Gene Set Enrichment Analysis of HPDL.** (**A**) The hallmarks gene set enrichment analysis (GSEA) of HPDL in pan-cancer. The size of the circle represents the false discovery rate (FDR) value of each cancer enrichment item, and the color represents the normalized enrichment score (NES) of each enrichment item. (**B**–**I**) HPDL’s GSEA results in TCGA pan-cancer. The most 20 related pathways of GSEA are presented in the form of a mountain map, and immune-related pathways are marked in red.

To uncover the biological mechanisms associated with HPDL expression in cancer, we conducted a comparative examination of gene expression. The examination involved comparing the subset that exhibited elevated HPDL levels to the subset that displayed low HPDL levels, with a particular emphasis on distinct forms of cancer. Supplementary Table 1 provides an overview of the genes showing differential expression (DEGs) in every type of cancer. Using the DEGs between the high and low-HPDL subgroups, we carried out GSEA analysis across 33 cancer types to assess HPDL-associated cancer hallmarks. A significant decrease in immune-related pathways, such as TNFA-signaling-via-NFKB, IFN-alpha response, IFN-gamma response, allograft rejection pathways, and inflammatory response, was observed, especially in different types of tumors, including BLCA, HNSC, KIRP, LUSC, OV, SKCM, and THCA. The results indicate a potential robust association between HPDL and the immune microenvironment of the tumor, along with the interplay between malignant cells and immune cells through ligand-receptor interactions. Furthermore, it was discovered that HPDL was linked to the transition from epithelial to mesenchymal in various types of tumors and displayed a notable inverse relationship with CESC, COAD, ESCA, GBM, HNSC, KIRP, LUSC, MESO, OV, READ, and STAD. This implies that HPDL could play a crucial role in the infiltration and mobility of cancerous growth. Additionally, oxidative phosphorylation, MYC targets, E2F targets, and G2M checkpoint exhibited a close association to HPDL expression in cancers (Figure 4A). To summarize, these findings provide proof that abnormal HPDL expression might have a role in the immune response toward tumors. Considerable studies have been carried out regarding the participation of HPDL in the growth and advancement of cancer, leading to subsequent exploration of its function in the immune response and the microenvironment of cancer.

GSEA was conducted to further explore the relationship between different pathways, specifically those related to the immune system, and HPDL in pancancer. Displayed in a mountain map format, Figure 4B–4I showed the top 20 associated pathways of GSEA, with immune-related pathways highlighted in red. HPDL was linked to the handling and exhibition of external peptide antigens, triggering T cells in the immune reaction, antigen processing-presentation, B-cell receptor signaling pathways, and reactions to chemokines in BLCA. The significance of HPDL in immune regulation is emphasized by these discoveries.

### Immune cell infiltration analyses of HPDL across cancers

**Figure 5:**
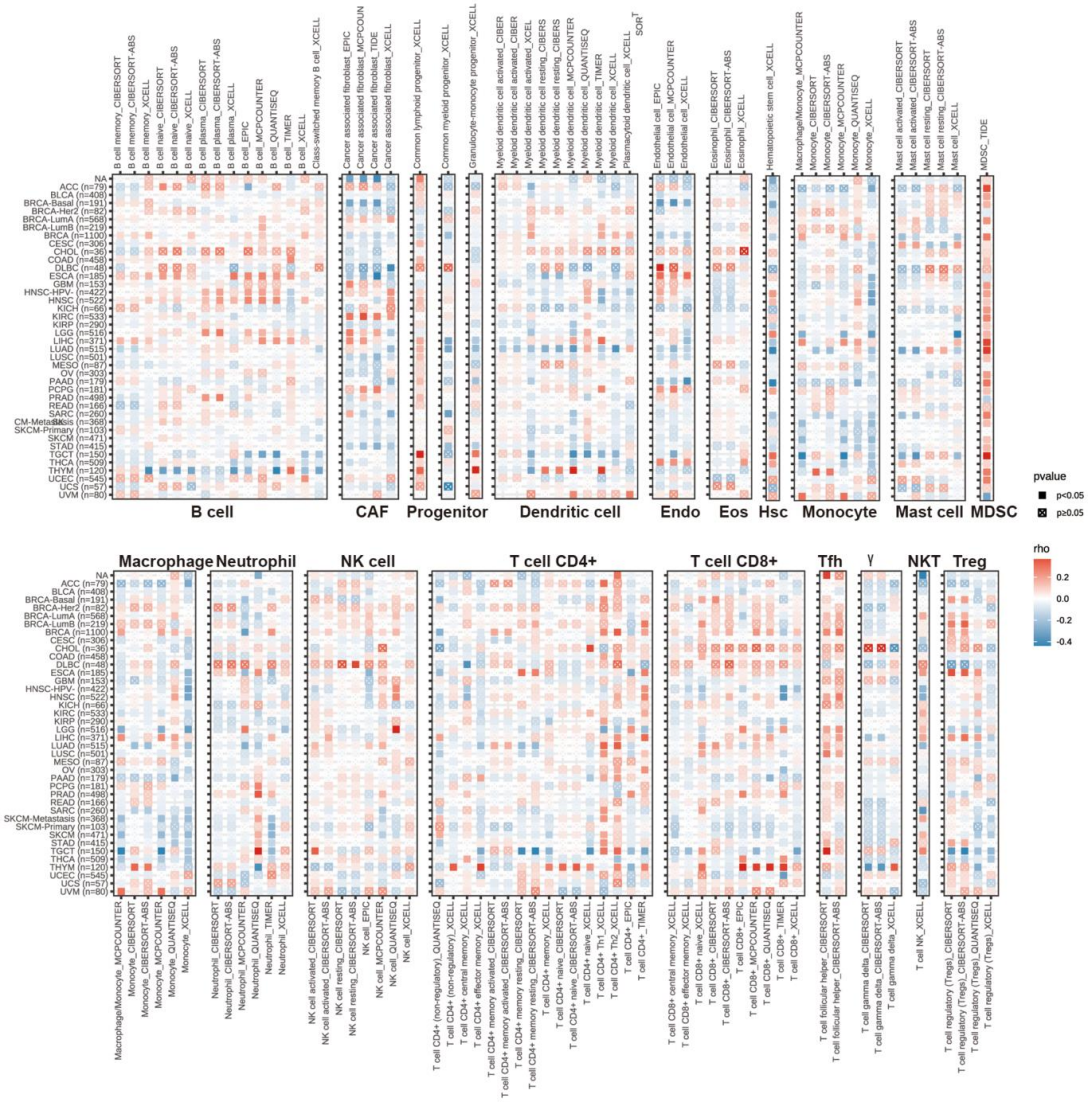
**The correlations of HPDL expression and the infiltration levels of CD4+ T cells, CAF, progenitor, Endo, Eos, HSC, Tfh, gdT, NKT, regulatory T cells (Tregs), B cells, neutrophils, monocytes, macrophages, dendritic cells, NK cells, Mast cells and CD8+ T cells in cancers.** Positive correlation in red and negative correlation in blue.

Following the analysis of the aforementioned findings, we explored the correlations between HPDL expression and the level of immune cell infiltration in different types of cancer. We utilized the TIMER2.0 database, which encompasses various quantitative platforms for investigating immune infiltration in cancer research, to exhibit the association between HPDL and the infiltration of immune cells. The findings indicate the levels of infiltration of various cell types, including CD4+ T cells, cancer-associated fibroblasts (CAFs), lymphoid progenitors, myeloid progenitors, endothelial cells (Endo), eosinophils (Eos), hematopoietic stem cells (HSCs), follicular helper T cells (Tfhs), gamma delta T cells (g/dT), natural killer T cells (NKTs), regulatory T cells (Tregs), myeloid-derived suppressor cells (MDSCs), neutrophils, monocytes, B cells, dendritic cells, macrophages, mast cells, NK cells, and CD8+ T cells, in pancancer (Figure 5).

Overall, the expression of HPDL showed a negative correlation with the level of immune infiltration from different types of infiltrating cells, including CAFs, lymphoid progenitors, myeloid progenitors, endo cells, HSCs, macrophages, mast cells, CD8+ T cells, CD4+ T cells, NKT cells, and g/dT cells, in various cancer forms. We observed a notable correlation between HPDL expression and a variety of infiltrating immune cells, including macrophages, B cells, cancer-associated fibroblasts (CAFs), and CD8+ T cells, in lung adenocarcinoma (LUAD), thymic carcinoma (THYM), and testicular germ cell tumors (TGCTs). Nevertheless, the association among these variables exhibited slight variances, possibly due to the varying degrees of immune infiltration in different tumors.

The results emphasize the intricate connection between HPDL expression and the infiltration of immune cells in various types of cancer, indicating that HPDL might have a role in regulating the immune microenvironment of tumors. This further emphasizes the significance of conducting additional research on the function of HPDL in the infiltration of immune cells and its potential consequences for cancer treatment and immunotherapy.

### Correlations between HPDL and immunomodulators, TMB, and MSI

**Figure 6:**
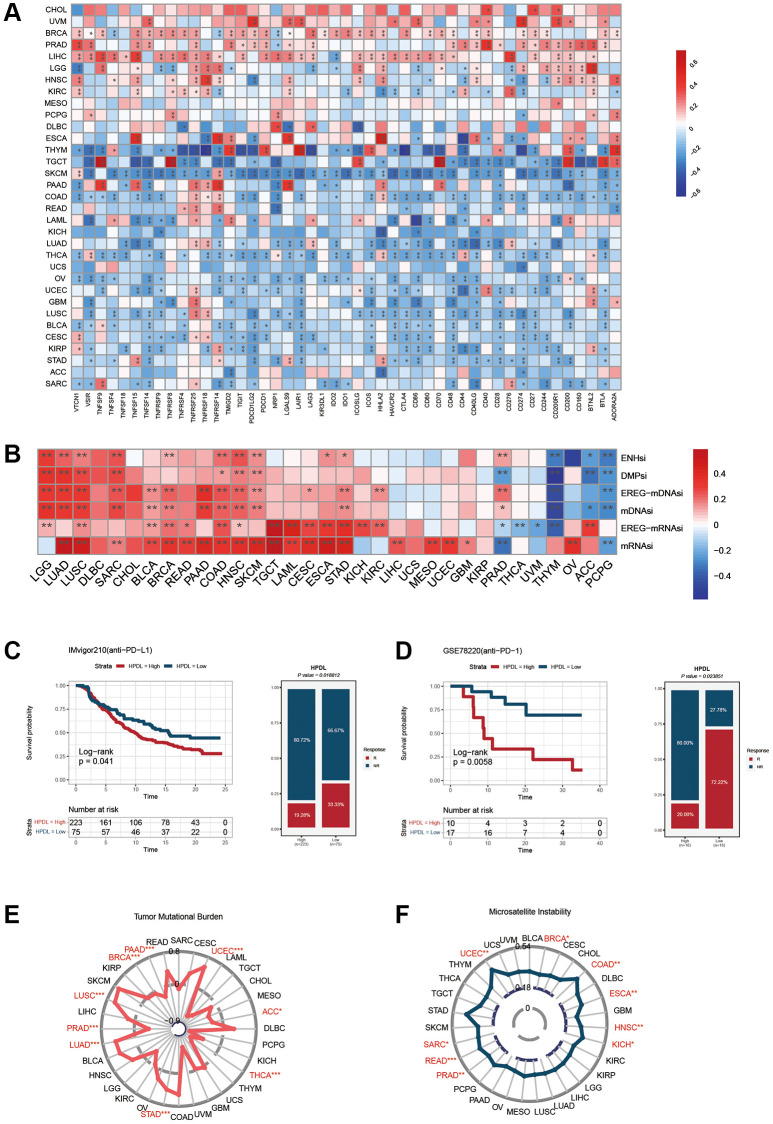
**Influence of HPDL expression on anti-tumor immunity and immunotherapy response.** (**A**) The Spearman correlation heatmap shows the correlation between the expression of HPDL and 47 kinds of immune regulators in pan-cancer. Red represents positive correlation and blue represents negative correlation. (**B**) The correlation between HPDL expression and the ENHsi, DMPsi, EREG-mDNAsi, mDNAsi, EREG-mRNAsi, and mRNAsi. (**C**) Kaplan-Meier curve of low and high-HPDL subgroup in IMvigor210 cohort (anti-PD-L1), and the proportion of tumors (including kidney cancer) in the low-HPDL and high-HPDL subgroups in the IMvigor210 cohort who responded to PD-1 therapy. (**D**) Kaplan-Meier curve of GSE78220 (anti-PD-1 melanoma) in the low and high-HPDL patient groups, and the proportion of melanoma patients in the GSE78220 low and high-HPDL subgroups that responded to anti-PD-1 therapy. (**E**, **F**) The correlation between HPDL expression and the TMB (**E**), and MSI (**F**). The labelled asterisk indicated the statistical *p*-value (^*^p < 0.05, ^**^p < 0.01, ^***^p < 0.001).

Spearman correlation analysis was used in Figure 6A to depict the investigation of the connections between HPDL expression and 47 immunomodulatory genes, encompassing immune checkpoint genes and immune cell marker genes, across various cancer types. In THYM, TGCT, SKCM, COAD, LUAD, THCA, OV, UCEC, GBM, LUSC, BLCA, CESC, KIRP, and STAD, we observed a negative correlation between HPDL and the majority of immunomodulatory factors. However, HPDL showed a positive correlation with most of these factors in BRCA, PRAD, and LIHC. Furthermore, HPDL exhibited a robust correlation with mRNAsi and mDNAsi in cases of LUAD, LUSC, SARC, BLCA, BRCA, PAAD, COAD, HNSC, SKCM, and STAD (Figure 6B). Conversely, it exhibited a negative relationship with mDNAsi in THYM, ACC, and PCPG. For BLCA, increased HPDL expression may cause a reduced response to immune checkpoint blockade therapy, while for PCPG, elevated HPDL expression may lead to increased vulnerability to immune checkpoint blockade treatment. It is worth mentioning that in PRAD, HPDL exhibited a favorable association with mRNAsi while displaying an unfavorable association with mDNAsi. The discrepancy between mRNAsi and mDNAsi, which could be attributed to DNA hypermethylation, may have led to this outcome [26].

The results offer valuable perspectives on the intricate connection between HPDL expression and different immunomodulatory factors, including TMB, MSI, and the index of tumor stemness. Understanding the function of HPDL in tumor immunity and its impact on the advancement of cancer immunotherapies can be facilitated by these valuable data.

### HPDL predicts the response to cancer immunotherapy

The use of immune checkpoint inhibitors (ICIs), including antibodies that specifically target PD-L1, PD-1, and CTLA-4 [27], has resulted in a substantial revolution in the field of cancer immunotherapy. After analyzing the previous results, we assessed the prognostic significance of HPDL in cancer patients who underwent ICI treatment.

Figure 6C displays the findings that suggest a robust correlation between HPDL expression and the response to treatment with anti-PD-L1. Patients exhibiting reduced HPDL expression demonstrate a superior rate of positive survival and longer duration compared to individuals with low HPDL expression. Individuals in the IMvigor210 group who had tumors in the urinary system and showed low HPDL levels had a response rate of 33.33% to anti-PD-L1 treatment, which was higher than the 19.28% response rate seen in individuals with high HPDL levels.

Similarly, melanoma patients treated with anti-PD-1 showed similar results. In the melanoma group GSE78220 (depicted in Figure 6D), individuals exhibiting reduced HPDL levels had a higher likelihood of survival than those with elevated HPDL expression. Moreover, among the individuals demonstrating reduced HPDL levels, PD-1 treatment proved to be effective in 72.22% of cases, whereas only 20% of patients with elevated HPDL expression responded positively to PD1 therapy. We also explored the relationship between HPDL and tumor mutation load and microsatellite instability. The results showed that the HPDL expressions of LUAD, LUSC, STAD, and UCEC were positively correlated with the TMB value and were negatively correlated with the TMB of BRCA, PAAD, PRAD, THCA, ACC, and THCA (Figure 6E). In addition, a positive correlation was found between the expressions of HPDL and MSI in BRCA, UCEC, SARC, READ, PRAD, HNSC, ESCA, COAD, and KICH (Figure 6F).

The data validate HPDL’s capacity to anticipate the response to immunotherapy and propose that HPDL may serve as a valuable marker for cancer immunotherapy. Nevertheless, considering the comprehensive outcomes, additional clinical and mechanistic research is needed to explore the prognostic significance of HPDL in various cancer types. Enhancing our comprehension of the function of HPDL in cancer immunotherapy holds the possibility of enhancing patient outcomes.

### Potential targeted drugs for HPDL expression

**Figure 7:**
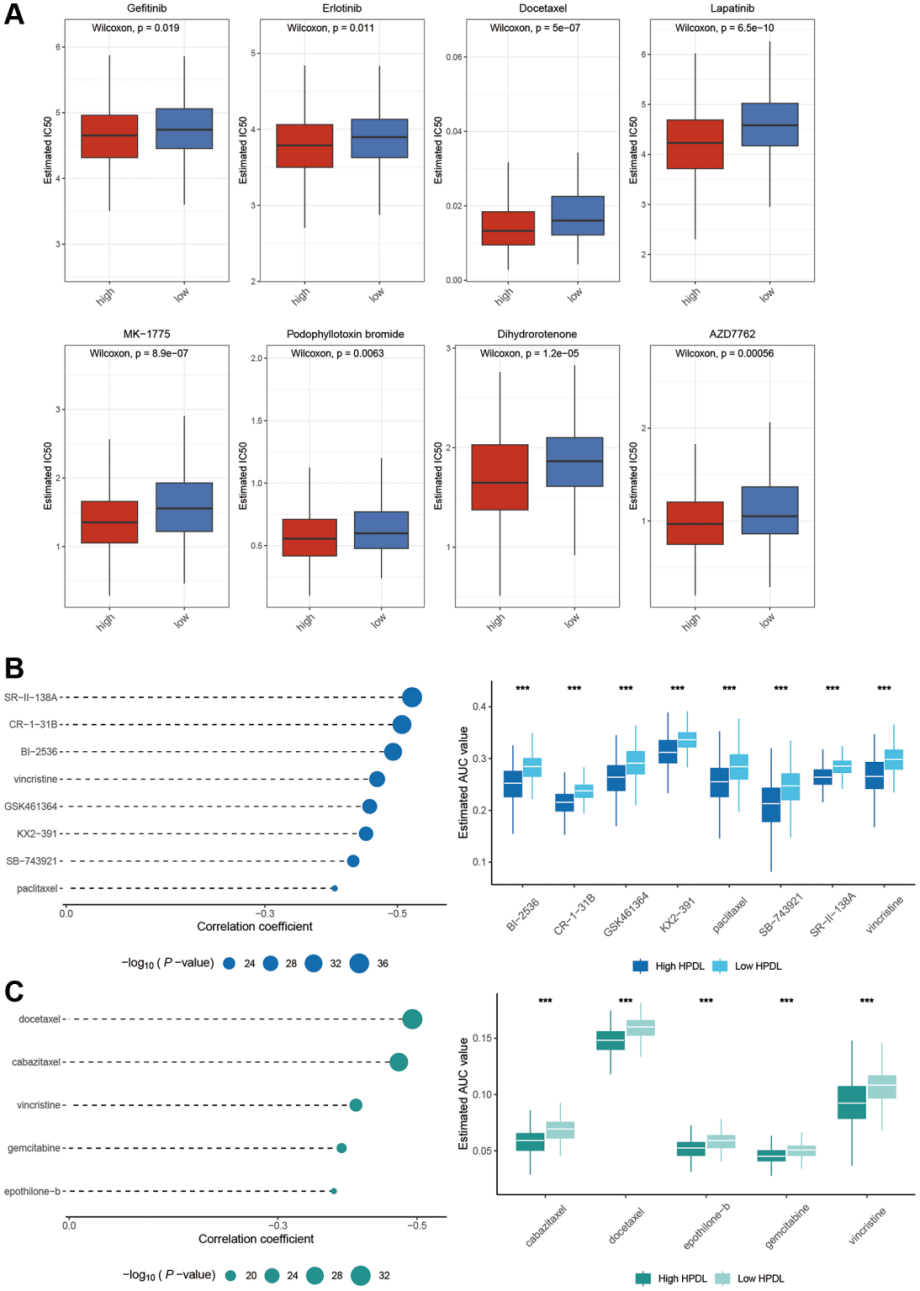
**Identification of candidate drugs based on the expression of HPDL.** (**A**) Estimated IC50 of the indicated molecular targeted drugs in High-HPDL and Low-HPDL group. (B, C) The results of correlation analysis and differential drug response analysis of CTRP (**B**) and PRISM (**C**) derived drugs in LUAD. The labelled asterisk indicated the statistical *p*-value (^*^p < 0.05, ^**^p < 0.01, ^***^p < 0.001).

On the basis of the aforementioned clues, we investigated the ability of HPDL to predict chemotherapy reactions in LUAD. Our research revealed that the High-HPDL group showed increased vulnerability to eight frequently used chemotherapy drugs, gefitinib, erlotinib, docetaxel, lapatinib, MK-1775, podophyllotoxin bromide, dihydrorotenone, and AZD7762 (Figure 7A).

To further elaborate on this analysis, we utilized gene expression and drug sensitivity information obtained from the CTRP and PRISM datasets to generate forecasts concerning potential drugs. A total of 1670 compounds remained after eliminating duplicates and excluding NA values. To select compounds, we established the threshold by considering the disparity in AUC values (log2FC > 0.1) between the high-HPDL and low-HPDL groups, along with the existence of negative correlation coefficients (r < −0.4) between HPDL expression and AUC. Figure 7B, 7C show that the examination of the CTRP database produced 8 drugs (SR-I-138A, CR-1-31B, BI-2536, vincristine, GSK461364, KX2-391, SB-743921, and paclitaxel), while the PRISM database revealed 5 medications (docetaxel, cabazitaxel, vincristine, gemcitabine, epothilone-b). LUAD can potentially be inhibited from HPDL activation by these medications.

### HPDL promote tumor growth, migration, and cell cycle in LUAD cells

**Figure 8:**
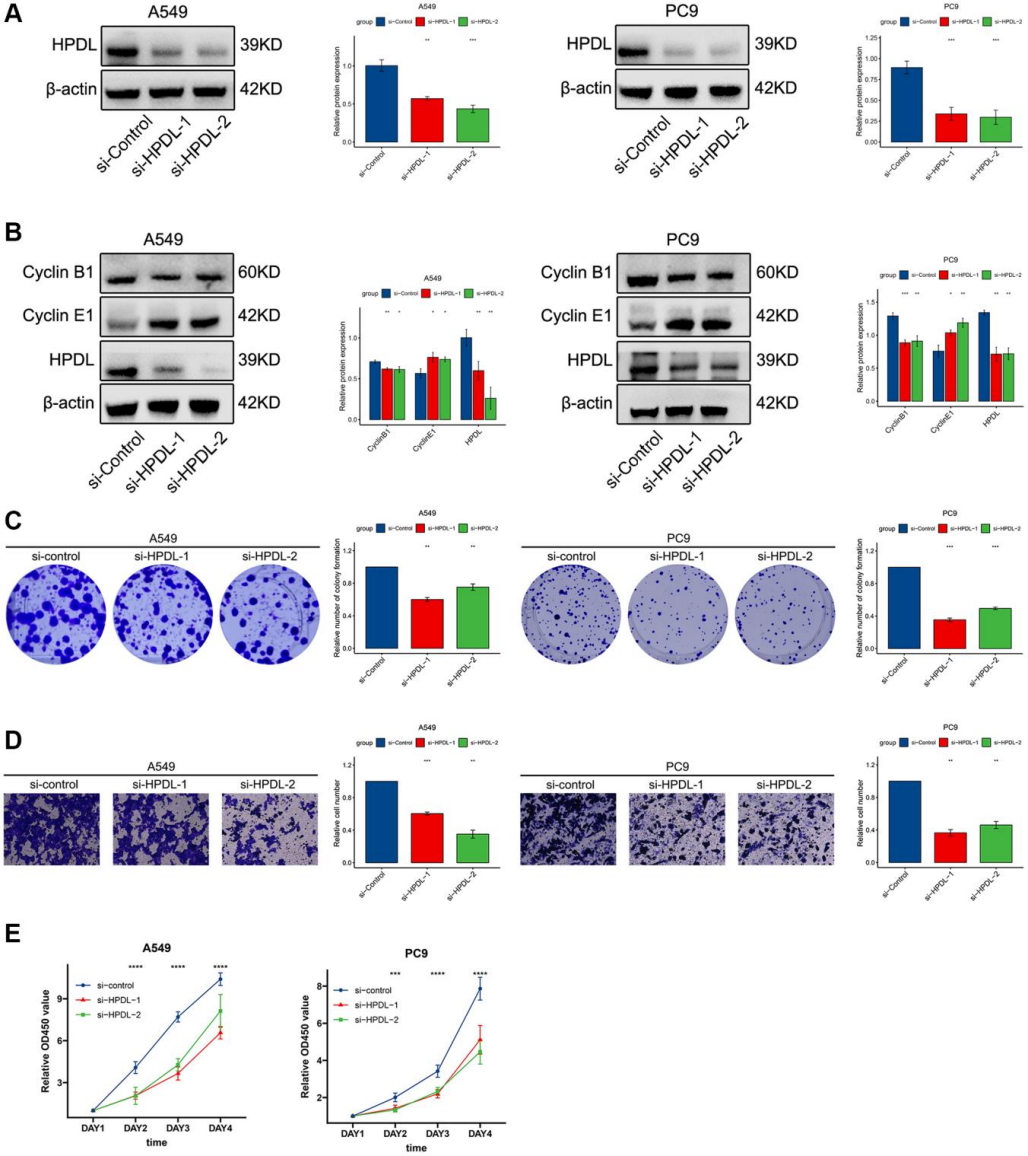
**HPDL promote tumor growth, migration, and cell cycle in LUAD cells.** (**A**) The level of HPDL transfection with siRNA were analyzed by WB. (**B**) The expression of Cyclin B1, Cyclin E1, and HPDL was analyzed by WB. (**C**) Colony formation assays showed that knockdown of HPDL inhibited LUAD cell growth. (**D**) Transwell assay showed that HPDL silencing inhibited the migrating of A549 and PC9 cells. (**E**) CCK8 assay was performed to determine the proliferation of HPDL knockdown. The labelled asterisk indicated the statistical *p*-value (^*^p < 0.05, ^**^p < 0.01, ^***^p < 0.001).

To investigate the role of HPDL, we utilized specific siRNAs to suppress gene expression in A549 and PC9 cells. Two siRNAs targeting the coding region of HPDL were tested for their knockdown efficiency (Figure 8A). According to WB analysis, the reduction in HPDL resulted in an increase in the expression of cyclin E1 while also causing a decrease in the expression of cyclin B1 (Figure 8B). From our prior analysis of gene sets, we observed a significant enrichment of the pathway associated with cell division. The abovementioned results validated that HPDL has the potential to regulate the cell cycle pathway in LUAD (Figure 8B).

Subsequently, we conducted colony formation and sphere formation experiments to assess the impact of HPDL on the proliferation of LUAD cells. According to the analysis of the colony, the ability to produce copies was significantly reduced following the inhibition of HPDL (Figure 8C). In line with these findings, the Transwell assay validated that inhibiting HPDL led to a decrease in the invasion of A549 and PC9 cells (Figure 8D). Afterwards, we examined the influence of HPDL on the progression of LUAD using CCK8. The results of our study showed that suppressing HPDL expression led to a decrease in the proliferation of A549 and PC9 cells (Figure 8E). The results suggest that HPDL may have a significant effect on the proliferation and invasion of LUAD cells.

## DISCUSSION

HPDL, a protein situated in the mitochondrial intermembrane space, mechanistically influences the three-dimensional growth of pancreatic ductal adenocarcinoma (PDAC) cell lines and promotes the proliferation of MIAPACA2 orthotopic xenografts [6, 7]. Additionally, overexpression of HPDL expedites PDAC growth *in vitro* [3]. However, our study focuses HPDL on immunotherapy, seeking new discoveries.

Initially, the levels of HPDL expression in pancancer were examined by utilizing the GTEx and TCGA databases. Figure 2A demonstrates that HPDL displayed abnormal upregulation in twenty-one cancer types and downregulation in six cancer types. Nevertheless, there was no correlation observed between HPDL and genetic mutations in tumors, as the proportion we observed was below 5% (Figure 2B). Based on our analysis, the increase in HPDL led to an increase in HPDL occurrences in OV patients, despite the limited genetic changes detected in other cancer forms (Figure 2C). The reason behind the uneven expression of HPDL in cancers remains unresolved. Overexpression of HPDL facilitates the development of tumors in pancreatic ductal adenocarcinoma (PDAC) cells [3]. The irregularity in the progression of the cell cycle is a vital process that drives the development of tumors, highlighting the significance of directing attention toward regulators of the cell cycle machinery for anticancer treatments [28]. The results obtained from GSEA indicate a strong association between HPDL expression and the G2M checkpoint in cancer, which aligns with this finding. Further investigation is needed to explore the abnormal expression of HPDL in cancer and its role in the process of cellular division. Afterwards, we assessed the correlation between HPDL and the clinical outcome of individuals diagnosed with cancer. The analysis findings of the operating system, DFI, DSS, and PFI in Figure 2A reveal a strong consensus among individuals with cancer, indicating a significant correlation between HPDL and the prognosis of cancer patients. To clarify, HPDL posed a risk to nine categories of people with cancer while offering protection against four types of cancer. According to the results, HPDL plays a vital role in predicting the future prospects of cancer patients and is expected to become a powerful indicator for the prognosis of cancer patients in the future.

In our analysis, the GSEA findings reveal a strong association between HPDL and immune response-related processes, including TNFA signaling via NFKB, IFN-alpha response, IFN-gamma response, and inflammatory response. Prior research has also emphasized that individuals with elevated HPDL levels in PDAC encounter unfavorable outcomes in terms of their overall survival. Consequently, treatments aimed at HPDL could provide advantages for these patients. However, understanding the variety of tumor dependence on HPDL is essential before these therapies can be advantageous [7]. The majority of these procedures experienced a notable reduction in the cancer subcategories exhibiting elevated HPDL levels, except for BRCA, CHOL, DLBC, ESCA, KIRC, LGG, PCPG, PRAD, and UVM. The results suggest that HPDL might have different roles in different types of cancer. In cancers, interferon (IFN)-γ-related mRNA profiles have been identified as predictive markers for chemotherapy resistance and the response of primary refractory/relapsed AML to flotetuzumab immunotherapy [29]. Similarly, a novel combination strategy involving IFNα and anti-PD-1 has shown efficacy in treating patients with Hepatocellular Carcinoma [30]. In the realm of immune response, TNF-α stands out as a major inflammatory factor, with anti-TNF-α antibodies revolutionizing the treatment landscape for numerous autoimmune disorders [31]. Furthermore, our study has shown that the inflammatory response, TNFA-signaling via NFKB, the IFN-a response, and the IFN-g response were closely related to the HPDL’s expression, providing a direction for further exploration of the role of HPDL in tumors. Our research yielded a noteworthy finding regarding the robust correlation between HPDL expression and the infiltration of immune cells in cancer, as depicted in Figure 4. HPDL showed a negative correlation with the infiltration of cancer-associated fibroblasts (CAFs), lymphoid progenitors, myeloid progenitors, endothelial cells (Endo cells), hematopoietic stem cells (HSCs), macrophages, mast cells, CD8+ T cells, CD4+ T cells, natural killer T cells (NKT cells), and gamma/delta T cells (g/dT cells) in the majority of tumors. This suggests that HPDL could impact the development and perspective of tumors through its influence on the surrounding tumor environment. In addition, the correlation analysis between HPDL and the pan-cancer immunomodulatory factors indicates that the HPDL expression was highly correlated to the expression of specific immunomodulatory genes (Figure 5), especially in CHOL, UVM, MESO, PCPG, and DLBC. The findings suggest that HPDL is likely involved in the advancement and outlook of cancer through its interaction with the microenvironment of the disease.

We next investigated the correlation between HPDL expression levels and the response to anti-PD-L1 immunotherapy in a pan-cancer cohort, including bladder cancer and the expression levels of anti-PD1 in melanoma (Figures 6C, 6D). In the IMvigor210 cancer cohort, it was found that a decrease in HPDL expression was associated with a favorable prognosis and improved response to anti-PD-L1 immunotherapy. Similar findings were observed in individuals with melanoma who received anti-PD-1 treatment (GSE78220 group). The group exhibiting elevated HPDL levels had a poorer prognosis and showed insensitivity to anti-PD-1 treatment. Nevertheless, the outcomes of the treatment for both individuals corresponded to their respective survival patterns. The findings indicate that HPDL acts as a reliable indicator for anticipating the reaction to immune checkpoint inhibition in different types of cancer. As a result, we speculated that HPDL might serve as a potent and encouraging indicator for forecasting the efficacy of cancer immunotherapy. During the antitumor immune response, the therapeutic efficacy largely depends on the functional status of tumor-specific effector immune cells [32–34]. Immune checkpoint costimulation and coinhibition signals regulate the multiplication and functioning of effector cells [35–37]. Consequently, the oncogenic immune response is often enhanced by promoting the costimulatory signal as positive or blocking the inhibitory signal of negative regulation [38]. Currently, numerous immune checkpoint molecules have been discovered to be applicable in medication treatment. PD-L1, PD-1, CTLA4, TIGIT, HAVCR2 (TIM-3 alias), and LAG-3 are the proteins that have been described [39–43]. The primary finding and thorough investigation have concentrated on CTLA4 and PD-L1/PD-1; the treatment of different cancers with immunotherapy frequently includes the combined utilization of anti-PD-1/PD-L1 and anti-CTLA-4. Nevertheless, the therapeutic outcome of this amalgamation lacks significance in numerous cancer patients and induces severe adverse reactions in patients. Hence, it is imperative to investigate novel immune checkpoints [44, 45] and methods for anticipating the efficacy of cancer immunotherapy. During this investigation, we discovered that HPDL exhibited great potential as a biomarker for predicting the outlook of various types of cancer. Furthermore, it has been shown to be a reliable measure for evaluating the effectiveness of immunotherapy in cancer treatment. The obtained findings offer crucial indications for future investigations into the possible involvement of HPDL in the immune response to tumors and immunotherapeutic strategies.

In summary, we have identified distinct barriers in the exploration of molecular targets that could result in novel anticancer inhibitors (Figure 7A–7C). Lapatinib, a tyrosine kinase inhibitor that targets EGFR and HER2 [46], enhances response rates and extends disease progression in patients with prior exposure to anthracycline-based and taxane-based chemotherapy and resistant tumors to trastuzumab [47]. Additionally, it improves the efficacy of capecitabine. Research has shown that dihydroartemisinin (DHA) has various molecular pathways that contribute to its anticancer properties. Several of these activities include restraining cell growth, triggering cell death, impeding the spread of tumors and formation of new blood vessels, enhancing the body's defense system, promoting self-degradation of cells, and causing stress to the endoplasmic reticulum [48]. Nevertheless, the specific process by which puromycin, lapatinib, DHA, and similar drugs related to HPDL impact the pancancer tumor microenvironment is still not understood. Further investigation is needed to explore the involvement of HPDL in the anticancer properties of these constituents. It is important to take into account the various constraints of our study. The exact relationship between HPDL and the elements identified through GDSC, CTRP, and PRISM datasets, as well as the potential mechanisms involved, is still unclear.

Moreover, the majority of our pancancer research data were acquired from publicly accessible online databases, potentially resulting in systematic bias due to the absence of comprehensive clinical cohort data for validation. Further experiments are necessary to clarify the HPDL mechanism’s impact on tumor occurrence and progression simultaneously. Furthermore, additional mechanistic studies are needed to elucidate and validate the potential medications that can be used in conjunction with HPDL and examined using CTRP and PRISM datasets.

## CONCLUSION

To summarize, we performed an extensive analysis of HPDL in various forms of cancer, demonstrating its capacity as a biomarker for cancer prognosis and its effectiveness in forecasting the reaction to immunotherapy. The unusual presentation of HPDL was linked to the immune regulation, infiltration of immune cells, tumor surroundings, TMB, and MSI of different tumor varieties. Our study establishes HPDL as a significant prognostic marker in clinical settings, indicating its utility not only in predicting cancer prognosis but also the efficacy of immunotherapies, thereby highlighting its potential as a target for immunotherapy. Suppression of HPDL expression resulted in reduced growth and movement of LUAD cells.

## Supplementary Materials

Supplementary Table 1

## References

[1] Callao V, Montoya E. Toxohormone-like factor from microorganisms with impaired respiration. Science. 1961; 134:2041–2. 10.1126/science.134.3495.2041 13875778

[2] Xia L, Oyang L, Lin J, Tan S, Han Y, Wu N, Yi P, Tang L, Pan Q, Rao S, Liang J, Tang Y, Su M, et al. The cancer metabolic reprogramming and immune response. Mol Cancer. 2021; 20:28. 10.1186/s12943-021-01316-8 33546704 PMC7863491

[3] Ye X, Wei X, Liao J, Chen P, Li X, Chen Y, Yang Y, Zhao Q, Sun H, Pan L, Chen G, He X, Lyu J, Fang H. 4-Hydroxyphenylpyruvate Dioxygenase-Like Protein Promotes Pancreatic Cancer Cell Progression and Is Associated With Glutamine-Mediated Redox Balance. Front Oncol. 2021; 10:617190. 10.3389/fonc.2020.617190 33537239 PMC7848781

[4] Johnson MO, Wolf MM, Madden MZ, Andrejeva G, Sugiura A, Contreras DC, Maseda D, Liberti MV, Paz K, Kishton RJ, Johnson ME, de Cubas AA, Wu P, et al. Distinct Regulation of Th17 and Th1 Cell Differentiation by Glutaminase-Dependent Metabolism. Cell. 2018; 175:1780–95.e19. 10.1016/j.cell.2018.10.001 30392958 PMC6361668

[5] Hildebrand D, Heeg K, Kubatzky KF. Pasteurella multocida Toxin Manipulates T Cell Differentiation. Front Microbiol. 2015; 6:1273. 10.3389/fmicb.2015.01273 26635744 PMC4652077

[6] Chen X, Chen S, Yu D. Metabolic Reprogramming of Chemoresistant Cancer Cells and the Potential Significance of Metabolic Regulation in the Reversal of Cancer Chemoresistance. Metabolites. 2020; 10:289. 10.3390/metabo10070289 32708822 PMC7408410

[7] Banh RS, Kim ES, Spillier Q, Biancur DE, Yamamoto K, Sohn ASW, Shi G, Jones DR, Kimmelman AC, Pacold ME. The polar oxy-metabolome reveals the 4-hydroxymandelate CoQ10 synthesis pathway. Nature. 2021; 597:420–5. 10.1038/s41586-021-03865-w 34471290 PMC8538427

[8] Doimo M, Desbats MA, Cerqua C, Cassina M, Trevisson E, Salviati L. Genetics of coenzyme q10 deficiency. Mol Syndromol. 2014; 5:156–62. 10.1159/000362826 25126048 PMC4112527

[9] Morgan NV, Yngvadottir B, O'Driscoll M, Clark GR, Walsh D, Martin E, Tee L, Reid E, Titheradge HL, Maher ER. Evidence that autosomal recessive spastic cerebral palsy-1 (CPSQ1) is caused by a missense variant in *HPDL*. Brain Commun. 2021; 3:fcab002. 10.1093/braincomms/fcab002 33634263 PMC7892364

[10] Husain RA, Grimmel M, Wagner M, Hennings JC, Marx C, Feichtinger RG, Saadi A, Rostásy K, Radelfahr F, Bevot A, Döbler-Neumann M, Hartmann H, Colleaux L, et al. Bi-allelic HPDL Variants Cause a Neurodegenerative Disease Ranging from Neonatal Encephalopathy to Adolescent-Onset Spastic Paraplegia. Am J Hum Genet. 2020; 107:364–73. 10.1016/j.ajhg.2020.06.015 32707086 PMC7413886

[11] Ghosh SG, Lee S, Fabunan R, Chai G, Zaki MS, Abdel-Salam G, Sultan T, Ben-Omran T, Alvi JR, McEvoy-Venneri J, Stanley V, Patel A, Ross D, et al. Biallelic variants in HPDL, encoding 4-hydroxyphenylpyruvate dioxygenase-like protein, lead to an infantile neurodegenerative condition. Genet Med. 2021; 23:524–33. 10.1038/s41436-020-01010-y 33188300

[12] Wiessner M, Maroofian R, Ni MY, Pedroni A, Müller JS, Stucka R, Beetz C, Efthymiou S, Santorelli FM, Alfares AA, Zhu C, Uhrova Meszarosova A, Alehabib E, et al, and Genomics England Research Consortium, PREPARE network. Biallelic variants in HPDL cause pure and complicated hereditary spastic paraplegia. Brain. 2021; 144:1422–34. 10.1093/brain/awab041 33970200 PMC8219359

[13] Goldman MJ, Craft B, Hastie M, Repečka K, McDade F, Kamath A, Banerjee A, Luo Y, Rogers D, Brooks AN, Zhu J, Haussler D. Visualizing and interpreting cancer genomics data via the Xena platform. Nat Biotechnol. 2020; 38:675–8. 10.1038/s41587-020-0546-8 32444850 PMC7386072

[14] Li C, Tang Z, Zhang W, Ye Z, Liu F. GEPIA2021: integrating multiple deconvolution-based analysis into GEPIA. Nucleic Acids Res. 2021; 49:W242–6. 10.1093/nar/gkab418 34050758 PMC8262695

[15] Haeussler M, Zweig AS, Tyner C, Speir ML, Rosenbloom KR, Raney BJ, Lee CM, Lee BT, Hinrichs AS, Gonzalez JN, Gibson D, Diekhans M, Clawson H, et al. The UCSC Genome Browser database: 2019 update. Nucleic Acids Res. 2019; 47:D853–8. 10.1093/nar/gky1095 30407534 PMC6323953

[16] Gao J, Aksoy BA, Dogrusoz U, Dresdner G, Gross B, Sumer SO, Sun Y, Jacobsen A, Sinha R, Larsson E, Cerami E, Sander C, Schultz N. Integrative analysis of complex cancer genomics and clinical profiles using the cBioPortal. Sci Signal. 2013; 6:pl1. 10.1126/scisignal.2004088 23550210 PMC4160307

[17] Uhlén M, Fagerberg L, Hallström BM, Lindskog C, Oksvold P, Mardinoglu A, Sivertsson Å, Kampf C, Sjöstedt E, Asplund A, Olsson I, Edlund K, Lundberg E, et al. Proteomics. Tissue-based map of the human proteome. Science. 2015; 347:1260419. 10.1126/science.1260419 25613900

[18] Veres DV, Gyurkó DM, Thaler B, Szalay KZ, Fazekas D, Korcsmáros T, Csermely P. ComPPI: a cellular compartment-specific database for protein-protein interaction network analysis. Nucleic Acids Res. 2015; 43:D485–93. 10.1093/nar/gku1007 25348397 PMC4383876

[19] Ritchie ME, Phipson B, Wu D, Hu Y, Law CW, Shi W, Smyth GK. limma powers differential expression analyses for RNA-sequencing and microarray studies. Nucleic Acids Res. 2015; 43:e47. 10.1093/nar/gkv007 25605792 PMC4402510

[20] Yu G, Wang LG, Han Y, He QY. clusterProfiler: an R package for comparing biological themes among gene clusters. OMICS. 2012; 16:284–7. 10.1089/omi.2011.0118 22455463 PMC3339379

[21] Hänzelmann S, Castelo R, Guinney J. GSVA: gene set variation analysis for microarray and RNA-seq data. BMC Bioinformatics. 2013; 14:7. 10.1186/1471-2105-14-7 23323831 PMC3618321

[22] Li T, Fu J, Zeng Z, Cohen D, Li J, Chen Q, Li B, Liu XS. TIMER2.0 for analysis of tumor-infiltrating immune cells. Nucleic Acids Res. 2020; 48:W509–14. 10.1093/nar/gkaa407 32442275 PMC7319575

[23] Hugo W, Zaretsky JM, Sun L, Song C, Moreno BH, Hu-Lieskovan S, Berent-Maoz B, Pang J, Chmielowski B, Cherry G, Seja E, Lomeli S, Kong X, et al. Genomic and Transcriptomic Features of Response to Anti-PD-1 Therapy in Metastatic Melanoma. Cell. 2016; 165:35–44. 10.1016/j.cell.2016.02.065 26997480 PMC4808437

[24] Yang C, Huang X, Li Y, Chen J, Lv Y, Dai S. Prognosis and personalized treatment prediction in TP53-mutant hepatocellular carcinoma: an in silico strategy towards precision oncology. Brief Bioinform. 2021; 22:bbaa164. 10.1093/bib/bbaa164 32789496

[25] Maeser D, Gruener RF, Huang RS. oncoPredict: an R package for predicting in vivo or cancer patient drug response and biomarkers from cell line screening data. Brief Bioinform. 2021; 22:bbab260. 10.1093/bib/bbab260 34260682 PMC8574972

[26] Malta TM, Sokolov A, Gentles AJ, Burzykowski T, Poisson L, Weinstein JN, Kamińska B, Huelsken J, Omberg L, Gevaert O, Colaprico A, Czerwińska P, Mazurek S, et al, and Cancer Genome Atlas Research Network. Machine Learning Identifies Stemness Features Associated with Oncogenic Dedifferentiation. Cell. 2018; 173:338–54.e15. 10.1016/j.cell.2018.03.034 29625051 PMC5902191

[27] Bagchi S, Yuan R, Engleman EG. Immune Checkpoint Inhibitors for the Treatment of Cancer: Clinical Impact and Mechanisms of Response and Resistance. Annu Rev Pathol. 2021; 16:223–49. 10.1146/annurev-pathol-042020-042741 33197221

[28] Liu J, Peng Y, Wei W. Cell cycle on the crossroad of tumorigenesis and cancer therapy. Trends Cell Biol. 2022; 32:30–44. 10.1016/j.tcb.2021.07.001 34304958 PMC8688170

[29] Vadakekolathu J, Minden MD, Hood T, Church SE, Reeder S, Altmann H, Sullivan AH, Viboch EJ, Patel T, Ibrahimova N, Warren SE, Arruda A, Liang Y, et al. Immune landscapes predict chemotherapy resistance and immunotherapy response in acute myeloid leukemia. Sci Transl Med. 2020; 12:eaaz0463. 10.1126/scitranslmed.aaz0463 32493790 PMC7427158

[30] Hu B, Yu M, Ma X, Sun J, Liu C, Wang C, Wu S, Fu P, Yang Z, He Y, Zhu Y, Huang C, Yang X, et al. IFNα Potentiates Anti-PD-1 Efficacy by Remodeling Glucose Metabolism in the Hepatocellular Carcinoma Microenvironment. Cancer Discov. 2022; 12:1718–41. 10.1158/2159-8290.CD-21-1022 35412588

[31] Andretto V, Dusi S, Zilio S, Repellin M, Kryza D, Ugel S, Lollo G. Tackling TNF-α in autoinflammatory disorders and autoimmune diseases: From conventional to cutting edge in biologics and RNA- based nanomedicines. Adv Drug Deliv Rev. 2023; 201:115080. 10.1016/j.addr.2023.115080 37660747

[32] Principe N, Kidman J, Goh S, Tilsed CM, Fisher SA, Fear VS, Forbes CA, Zemek RM, Chopra A, Watson M, Dick IM, Boon L, Holt RA, et al. Tumor Infiltrating Effector Memory Antigen-Specific CD8^+^ T Cells Predict Response to Immune Checkpoint Therapy. Front Immunol. 2020; 11:584423. 10.3389/fimmu.2020.584423 33262762 PMC7688517

[33] Wang Y, Xiang Y, Xin VW, Wang XW, Peng XC, Liu XQ, Wang D, Li N, Cheng JT, Lyv YN, Cui SZ, Ma Z, Zhang Q, Xin HW. Dendritic cell biology and its role in tumor immunotherapy. J Hematol Oncol. 2020; 13:107. 10.1186/s13045-020-00939-6 32746880 PMC7397618

[34] Knutson KL, Disis ML. Tumor antigen-specific T helper cells in cancer immunity and immunotherapy. Cancer Immunol Immunother. 2005; 54:721–8. 10.1007/s00262-004-0653-2 16010587 PMC11032889

[35] Mahoney KM, Freeman GJ, McDermott DF. The Next Immune-Checkpoint Inhibitors: PD-1/PD-L1 Blockade in Melanoma. Clin Ther. 2015; 37:764–82. 10.1016/j.clinthera.2015.02.018 25823918 PMC4497957

[36] Wei SC, Levine JH, Cogdill AP, Zhao Y, Anang NAS, Andrews MC, Sharma P, Wang J, Wargo JA, Pe'er D, Allison JP. Distinct Cellular Mechanisms Underlie Anti-CTLA-4 and Anti-PD-1 Checkpoint Blockade. Cell. 2017; 170:1120–33.e17. 10.1016/j.cell.2017.07.024 28803728 PMC5591072

[37] Giles AJ, Hutchinson MND, Sonnemann HM, Jung J, Fecci PE, Ratnam NM, Zhang W, Song H, Bailey R, Davis D, Reid CM, Park DM, Gilbert MR. Dexamethasone-induced immunosuppression: mechanisms and implications for immunotherapy. J Immunother Cancer. 2018; 6:51. 10.1186/s40425-018-0371-5 29891009 PMC5996496

[38] Gotwals P, Cameron S, Cipolletta D, Cremasco V, Crystal A, Hewes B, Mueller B, Quaratino S, Sabatos-Peyton C, Petruzzelli L, Engelman JA, Dranoff G. Prospects for combining targeted and conventional cancer therapy with immunotherapy. Nat Rev Cancer. 2017; 17:286–301. 10.1038/nrc.2017.17 28338065

[39] Okazaki T, Chikuma S, Iwai Y, Fagarasan S, Honjo T. A rheostat for immune responses: the unique properties of PD-1 and their advantages for clinical application. Nat Immunol. 2013; 14:1212–8. 10.1038/ni.2762 24240160

[40] Lee J, Choi Y, Jung HA, Lee SH, Ahn JS, Ahn MJ, Park K, Sun JM. Outstanding clinical efficacy of PD-1/PD-L1 inhibitors for pulmonary pleomorphic carcinoma. Eur J Cancer. 2020; 132:150–8. 10.1016/j.ejca.2020.03.029 32371248

[41] Mayes PA, Hance KW, Hoos A. The promise and challenges of immune agonist antibody development in cancer. Nat Rev Drug Discov. 2018; 17:509–27. 10.1038/nrd.2018.75 29904196

[42] Qin S, Xu L, Yi M, Yu S, Wu K, Luo S. Novel immune checkpoint targets: moving beyond PD-1 and CTLA-4. Mol Cancer. 2019; 18:155. 10.1186/s12943-019-1091-2 31690319 PMC6833286

[43] Rotte A, Jin JY, Lemaire V. Mechanistic overview of immune checkpoints to support the rational design of their combinations in cancer immunotherapy. Ann Oncol. 2018; 29:71–83. 10.1093/annonc/mdx686 29069302

[44] Naidoo J, Page DB, Li BT, Connell LC, Schindler K, Lacouture ME, Postow MA, Wolchok JD. Toxicities of the anti-PD-1 and anti-PD-L1 immune checkpoint antibodies. Ann Oncol. 2015; 26:2375–91. 10.1093/annonc/mdv383 26371282 PMC6267867

[45] Ramos-Casals M, Brahmer JR, Callahan MK, Flores-Chávez A, Keegan N, Khamashta MA, Lambotte O, Mariette X, Prat A, Suárez-Almazor ME. Immune-related adverse events of checkpoint inhibitors. Nat Rev Dis Primers. 2020; 6:38. 10.1038/s41572-020-0160-6 32382051 PMC9728094

[46] Xia W, Mullin RJ, Keith BR, Liu LH, Ma H, Rusnak DW, Owens G, Alligood KJ, Spector NL. Anti-tumor activity of GW572016: a dual tyrosine kinase inhibitor blocks EGF activation of EGFR/erbB2 and downstream Erk1/2 and AKT pathways. Oncogene. 2002; 21:6255–63. 10.1038/sj.onc.1205794 12214266

[47] Geyer CE, Forster J, Lindquist D, Chan S, Romieu CG, Pienkowski T, Jagiello-Gruszfeld A, Crown J, Chan A, Kaufman B, Skarlos D, Campone M, Davidson N, et al. Lapatinib plus capecitabine for HER2-positive advanced breast cancer. N Engl J Med. 2006; 355:2733–43. 10.1056/NEJMoa064320 17192538

[48] Dai X, Zhang X, Chen W, Chen Y, Zhang Q, Mo S, Lu J. Dihydroartemisinin: A Potential Natural Anticancer Drug. Int J Biol Sci. 2021; 17:603–22. 10.7150/ijbs.50364 33613116 PMC7893584

